# Correction: Dong et al. Smurf1 Suppression Enhances Temozolomide Chemosensitivity in Glioblastoma by Facilitating PTEN Nuclear Translocation. *Cells* 2022, *11*, 3302

**DOI:** 10.3390/cells13181575

**Published:** 2024-09-19

**Authors:** Lei Dong, Yang Li, Liqun Liu, Xinyi Meng, Shengzhen Li, Da Han, Zhenyu Xiao, Qin Xia

**Affiliations:** School of Life Science, Beijing Institute of Technology, Beijing 100081, China; ldong@bit.edu.cn (L.D.);

## Errors in Figures 1e and 3c

In the original publication [1], there were errors in Figures 1e and 3c as published. The β-actin of U343 in Figure 1e is incorrect; the β-actin of U251 was mistakenly placed in position U343 due to improper movement during the image organization. The images in Figure 3c are incorrect; the images were inadvertently inserted because the sample results of LN229 and LN229R were mistakenly placed in the same folder. We made the following corrections for Figure 1e and Figure 3c.

The authors state that the scientific conclusions are unaffected. This correction was approved by the Academic Editor. The original publication has also been updated.

## Figures and Tables

**Figure 1:**
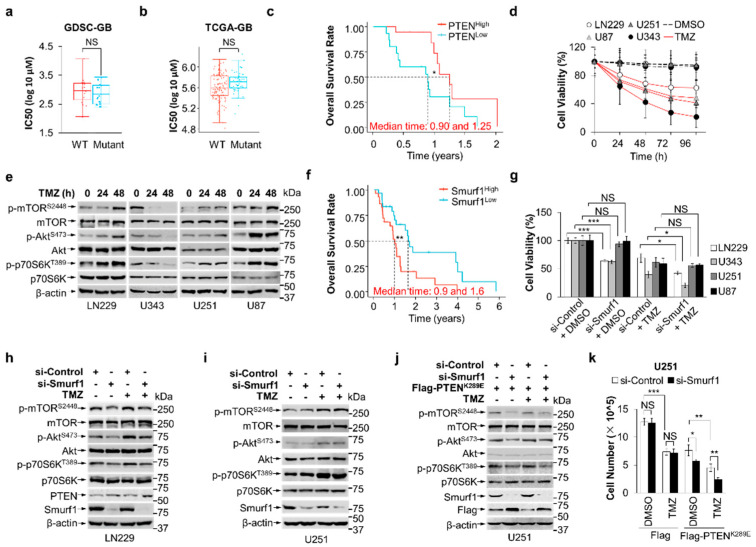
Combination of Smurf1 suppression with TMZ synthetically inhibits PI3K/Akt pathway. (**a**) The distribution of IC50 scores for TMZ in different-PTEN-status GB cell lines. The IC50 for TMZ and PTEN status of cell lines was obtained from the largest publicly available pharmacogenomics database (the Genomics of Drug Sensitivity in Cancer (GDSC), https://www.cancerrxgene.org/ (accessed on 19 September 2022)). PTEN wild-type group (wild type) included 8-MGBA, DBTRG05MG, SF268, GB-1, AM38, KS1, SNB75, YH13, LN18, LN229, DKMG, SW1088, U343, T98G, Becker, and SK-MG-1; PTEN mutant group (mutant) included U87, 42-MGBA, M059K, SF126, CAS1, U251, YKG1, U118, SF295, LN405, D263, LNZTA3WT4, D392-MG, D-542MG, D-247MG, D-566MG, and D-423MG. (**b**) The distribution of IC50 scores for TMZ in different-PTEN-status GB patients. RNA-sequencing expression (level 3) profiles and corresponding clinical information for GB cases were downloaded from the TCGA dataset (https://portal.gdc.com (accessed on 20 September 2022)). The GB cases were divided into PTEN wild-type group (wild type, *n* = 102) and PTEN mutant group (mutant, *n* = 47). Predicted the chemotherapeutic response for each sample based on GDSC. The prediction process was implemented by R package “pRRophetic”. The samples’ half–maximal inhibitory concentration (IC50) was estimated by ridge regression. All parameters were set as the default values. Using the batch effect of combat and tissue type of all tissues, the duplicate gene expression was summarized as mean value. The statistical difference between two groups was compared through the Wilcoxon test. (**c**) Kaplan-Meier survival analysis of the gene signature from TCGA dataset; comparison among different groups was conducted by log-rank test (* *p* = 0.034). The timeROC (v 0.4) analysis was used to compare the predictive accuracy of PTEN mRNA. Median time represents the time corresponding to the survival rate of 50% (i.e., the median survival time) in the high-expression group (1.25 years) and the low-expression group (0.9 years). (**d**) Cell viability was measured after the indicated time treatment with DMSO or TMZ (250 μM) in different GB cells (LN229, U87, U251, and U343) using MTT assay. (**e**) LN229, U343, U251, and U87 were cultured in the absence or presence of TMZ for the indicated time. The whole-cell lysates were examined through Western blotting for the expression of p-mTOR^S2448^/mTOR, p-Akt^S473^/Akt, p-p70S6K^T389^/p70S6K, and β-actin proteins. (**f**) Kaplan–Meier survival analysis of the gene signature from TCGA dataset; comparison among different groups was conducted by log-rank test (** *p* = 0.00902). The timeROC (v 0.4) analysis was used to compare the predictive accuracy of Smurf1 mRNA. Median time represents the time corresponding to the survival rate of 50% (i.e., the median survival time) in the high-expression group (0.9 years) and the low-expression group (1.6 years). All the analysis methods and R packages were implemented by R (foundation for statistical computing 2020) version 4.0.3. (**g**) MTT assay revealed the effect of Smurf1 knockdown on LN229, U343, U251, and U87 cells with DMSO or TMZ (250 μM, 48 h) treatment. (**h**,**i**) LN229 (**h**) and U251 (**i**) were transfected with si-Control or si-Smurf1 and then treated with DMSO or TMZ (250 μM, 48 h). Western blotting was employed to detect target proteins p-mTOR^S2448^/mTOR, p-Akt^S473^/Akt, p-p70S6K^T389^/p70S6K, PTEN, Smurf1, and β-actin. (**j**) U251 with Flag-PTEN^K289E^ was transfected with si-Control or si-Smurf1 and then treated with DMSO or TMZ (250 μM, 48 h). Western blotting was employed to detect target proteins p-mTOR^S2448^/mTOR, p-Akt^S473^/Akt, p-p70S6K^T389^/p70S6K, Flag, Smurf1, and β-actin proteins. (**k**) U251 with Flag or Flag-PTEN^K289E^ was transfected with si-Control or si-Smurf1 and then treated with DMSO or TMZ (250 μM, 48 h). The number of cells in each group were counted. In (**e**,**h**–**j**), Western blot analysis was performed in *n* = 3 biological replicates. In (**d**,**g**,**k**), data are presented as mean ± SD of three separate experiments, NS *p* > 0.05, * *p* < 0.05, ** *p* < 0.01, *** *p* < 0.001 as determined by unpaired two–tailed Student’s *t*-test.

**Figure 3:**
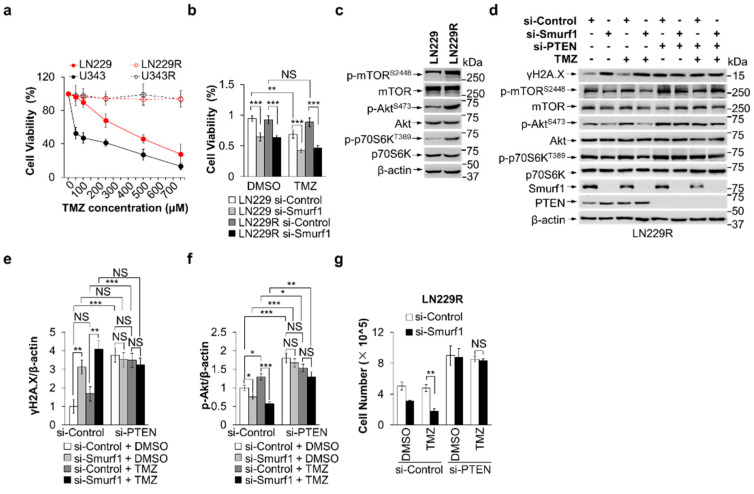
Smurf1 suppression restores the sensitivity of TMZ-resistant GB cells. (**a**) Cell viability was measured after 48 h treatment with increasing concentrations of TMZ in LN229, U343, LN229R, and U343R using MTT assay. (**b**) LN229 and LN229R were transfected with si-Control or si-Smurf1 followed by DMSO or TMZ (250 μM, 48 h) treatment. MTT assay was performed to evaluate the cytotoxicity of treatments on each group. Values were normalized against the amount of LN229 and LN229R treated with control siRNA oligos and DMSO. (**c**) LN229 and LN229R were prepared for whole-cell lysates and examined by immunoblotting with anti-p-mTOR^S2448^, anti-mTOR, anti-p-Akt^S473^, anti-Akt, anti-p-p70S6K^T389^, and anti-p70S6K antibodies. Blots were probed with anti-β-actin antibody to check protein loading. (**d**–**g**) LN229R cells were transfected with control, Smurf1, or PTEN siRNA oligos, followed by DMSO or TMZ (250 μM, 48 h) treatment. Western blotting was employed to detect target proteins γH2A.X, p-mTOR^S2448^, mTOR, p-Akt^S473^, Akt, p-p70S6K^T389^, p70S6K, Smurf1, PTEN, and β-actin (**d**). The graph shows the relative γH2A.X (**e**) and p-Akt (**f**) intensities. Values were normalized against the amount of LN229R treated with control siRNA oligos and DMSO. The number of cells in each group was counted (**g**). In (**c**,**d**), Western blot analysis was performed in *n* = 3 biological replicates. In (**a**,**b**,**e**,**f**,**g**), data are presented as mean ± SD of three separate experiments, NS *p* > 0.05, * *p* < 0.05, ** *p* < 0.01, *** *p* < 0.001 as determined by unpaired two-tailed Student’s *t*-test.
